# Ensuring Justification for Head CT Scans in Patients With Head Injuries According to 2023 National Institute of Excellence Guidelines at a Tertiary Care Center in Baghdad, Iraq: A Clinical Audit

**DOI:** 10.7759/cureus.95892

**Published:** 2025-11-01

**Authors:** Sarmad Al-Hilfi

**Affiliations:** 1 Department of Surgery, College of Medicine, University of Basra, Basra, IRQ

**Keywords:** clinical audit, ct scan head, emergency neurosurgery, guideline adherence, neuroimaging, radiology, traumatic brain injury

## Abstract

Objective

To evaluate adherence to the National Institute for Health and Care Excellence (NICE) guidelines in ordering computed tomography (CT) head scans for patients presenting with head trauma at the emergency department at the Teaching Hospital in Baghdad.

Methodology

A clinical audit was conducted in two cycles at the Radiology Department of the Teaching Hospital. Data were collected from the head CT request forms for patients presenting with head trauma to the emergency room. In the first cycle, data were gathered retrospectively over a month in January 2025. The data were then examined and analyzed, and interventions were implemented to enhance quality and outcomes. The second cycle was conducted to objectively evaluate the success of these interventions over a one-month period (May 2025).

Results

In cycle one, 144 patients were included, with 77.08% of their requests failing to comply with the NICE guidelines. After implementing the interventions, 28.44% of the 102 patients in the second cycle showed no definitive indications. The interventions significantly reduced requests that fell outside of the NICE guidelines.

Conclusion

The adherence to NICE guidelines for justifying head CT scans for head injury was inadequate and was significantly improved after implementing simple interventions.

## Introduction

A traumatic brain injury, or TBI, is an injury that impacts how the brain functions. It may be caused by a bump, blow, or jolt to the head or from a penetrating injury, such as from a gunshot, to the head. There are three main types of TBI: mild TBI, or concussion; moderate TBI; and severe TBI [1]. TBI presents a major global health challenge. Each year, more than 50 million people experience TBI, making up 30%-40% of all injury-related deaths worldwide [2]. Additionally, it costs the global economy around US$400 billion each year [3].

Imaging plays a crucial role in managing patients with TBI. Computed tomography (CT) scans are the preferred imaging technique in cases of acute head trauma, enabling precise detection and treatment of extra- and intra-axial hemorrhages, hydrocephalus, mass effect, and vascular injuries. CT scans are also effective in identifying secondary injuries and are therefore vital in follow-up [4,5].

For patients showing obvious signs of TBI, such as skull fracture evidence on physical exam or neurological changes, performing a head CT scan offers clear benefits because advanced imaging might be needed to guide medical and neurosurgical interventions. However, for patients without obvious signs of TBI, deciding whether to perform a head CT requires more careful consideration [6]. A study indicates that in the UK and USA, over 90% of patients with head trauma seen in emergency departments are diagnosed with minor head injuries, and CT scans have been consistently overused for patients with minor injuries, with studies revealing that a third of these scans could potentially be avoided [7]. In the Middle East and North Africa (MENA) region, mild TBI contributes significantly to the overall incidence compared to moderate and severe TBI cases [8].

A strategy of imaging all patients with a head injury guarantees not missing any clinically relevant structural damage, but it would be costly, expose many patients to unnecessary radiation, and increase crowding in the emergency department [9,10]. Moreover, sufficient clinical details should be provided to justify performing the diagnostic imaging exam. The threshold for justifying a medical imaging exam is that the expected clinical benefit outweighs the risks, including radiation exposure [11]. Therefore, a balanced approach is needed to order head CTs only when necessary and when clear head injury risk factors are present, while reducing the downsides of over-imaging [6]. This balance has been achieved through several clinical decision rules and guidelines, such as the Canadian CT Head Rule [12] and the UK National Institute for Health and Care Excellence (NICE) guidelines for head injury [13].

Although in Iraq, no national guideline is designated for TBI imaging, emergency medicine doctors often rely on the National Institute for Health and Care Excellence (NICE) guideline to justify performing a CT head scan for patients with TBI. This study aims to evaluate and improve adherence to NICE guidelines to justify performing CT head scans for patients with head trauma at the Teaching Hospital in Baghdad, Iraq.

## Materials and methods

Study design and data collection

This study was conducted in two cycles on patients presenting with head injuries in the emergency department (ED) who underwent a CT head scan at the Radiology Department during January and May 2025. Data were collected retrospectively from paper-based head CT scan request forms. Both clinical information, including the reason for examination (indications), and demographic data were recorded.

Formal ethical approval is not required for clinical audits according to the guidelines of the Ministry of Health in Iraq. However, the radiology department at the hospital approved to ensure ethical compliance and adherence to institutional protocols. We used IBM SPSS Statistics for Windows, Version 29.0.2.0 (IBM Corp., Armonk, New York, USA), to analyze the data, applying descriptive statistical methods such as percentages and means. The Chi-square test was used to assess the significance of associations, with a p-value of less than 0.05 set as the threshold for statistical significance.

Inclusion criteria

Any patient presenting with head trauma and aged 16 or older.

Exclusion criteria

Any patient presenting with non-trauma brain problems. The non-trauma brain case includes any central nervous system disease not caused by trauma, such as stroke, CNS infection, or tumor. Or anyone under 16 years old, even if they have head trauma.

Methods

The standard for this audit is the criteria that justify a head CT scan for people 16 years old and older who have sustained a head injury, in accordance with the NICE guidelines (NG232) [13]. Table 1 shows the NICE criteria.

**Table 1:** NICE criteria (NG232) for doing a CT head scan: for people 16 years old and over who have sustained a head injury. GCS: Glasgow Coma Scale; NICE: National Institute for Health and Care Excellence.

Do a CT head scan within one hour of any of these risk factors being identified	For people 16 and over who have had some loss of consciousness or amnesia since the injury, do a CT head scan within eight hours of the head injury
A GCS score of 12 or less on initial assessment in the emergency department	Age 65 or over
A GCS score of less than 15 at two hours after the injury on assessment in the emergency department	Any current bleeding or clotting disorders
Suspected open or depressed skull fracture	Dangerous mechanism of injury (a pedestrian or cyclist struck by a motor vehicle, an occupant ejected from a motor vehicle or a fall from a height of more than 1 m or five stairs)
Any sign of basal skull fracture (hemotympanum, 'panda' eyes, cerebrospinal fluid leakage from the ear or nose, Battle's sign)	More than 30 minutes' retrograde amnesia of events immediately before the head injury.
Post-traumatic seizure	
Focal neurological deficit	
More than one episode of vomiting	

The target is that all request forms for head injury cases must include clear documentation of the head injury risk factor(s) justifying the head CT scan.

Audit cycles

In the first cycle of the audit, the total sample consisted of 545 CT brain request forms from patients presented to the ER over one month (January 2025), with only 144 scans meeting our inclusion criteria (any patient presenting with head trauma and aged 16 or older). This sample size exceeds the minimum number recommended by the Royal College of Radiologists for such studies [14]. During the analysis of the first-cycle data, the audit team, composed of residents from emergency and radiology departments and a consultant radiologist, identified two potential factors that could influence adherence to NICE guidelines. The first factor concerns the doctor's knowledge and adherence to established guidelines, such as NICE guidelines, for managing head trauma. The second factor concerns the proper completion of request forms, specifically regarding the inclusion of adequate clinical information.

To improve adherence to the NICE guidelines, the audit team identified key strategies based on a literature review and input from the department. The first strategy is educating staff about the topic by sharing audit findings with ED staff during a team meeting. The second strategy is adopting the 2023 NICE guidelines as the standard protocol for TBI management in the hospital. The third strategy involves using a new radiology request form with a checklist of indications from NICE guidelines. The fourth strategy involves providing ongoing staff education through targeted sessions and follow-up practice.

Regarding the proper completion of the radiology request, it is recommended that the request form include sufficient clinical details, such as relevant clinical history, the primary clinical diagnosis being questioned, and clinical signs, to justify the performance of the diagnostic imaging examination [15-17]. This aligns with the protocol for requesting a CT scan at the hospital's radiology department. Therefore, the adequacy of clinical information was evaluated in cycle one to determine if it influenced the documentation of clear head injury risk factors (indications) justifying the scan and whether it could serve as an additional strategy.

After implementing the proposed interventions, a second audit cycle (cycle two) was conducted over a 30-day period in May 2025 to assess their impact. Of the 456 CT scans reviewed, 102 met the inclusion criteria.

## Results

During the analysis of patient demographics, the mean age of cycle one patients was 39.14±16.88 years, with the youngest being 16 years old and the oldest being 81 years old. The study population consisted of 73.6% male patients. In the second cycle, the study population had a mean age of 38.04 ± 16.45 years. The youngest patient was 16 years old, and the oldest was 82 years old. Male patients continued to predominate, accounting for 70.58% of the total. This demographic information is shown in Tables 2, 3.

**Table 2:** The demographics of patients in cycle one. The data are presented as n (%).

Cycle one	Total patients	Male patients	Female patients
Number and percentage	144	106 (73.6%)	38 (26.4%)
Average age (years) (mean±SD)	39.14±16.88	38.23±17.45	41.69±15.32

**Table 3:** The demographics of patients in cycle two. The data are presented as n (%).

Cycle two	Total patients	Male patients	Female patients
Number and percentage	102	72 (70.58%)	30 (29.41%)
Average age (years) (mean±SD) age	38.04±16.45	37.44±17.33	39.48±14.33

Regarding adherence to NICE guidelines, in the first cycle, we found that 33 request forms met the NICE criteria for both one and eight hours, representing 22.91% of the scans. In contrast, 111 scans had no clear indications, accounting for 77.08% of the scans. In comparison, during cycle two, 73 request forms met the NICE criteria, constituting 71.56% of the scans, while only 29 scans did not meet the criteria, making up 28.44%. This indicates a 29.16% decrease in the total number of head CT scans for head trauma per month due to a reduction in unnecessary scans and a 48.56% reduction in scans outside the NICE criteria after implementing the intervention. This significant association is detailed in Table 4, with a p-value less than 0.05.

**Table 4:** Comparison of general adherence to NICE criteria between the two cycles. The data are presented as n (%). Statistical analysis was performed using the Chi-square test, with significance set at p<0.05. Chi-square value (χ²) = 57.63. NICE: National Institute for Health and Care Excellence.

Cycle	Total scans	Scans adherent to the NICE guidelines	Scans not adherent to the guidelines	P-value
Cycle one	144	33 (22.91%)	111 (77.08%)	<0.0001
Cycle two	102	73 (71.56%)	29 (28.44%)	

**Figure 1:**
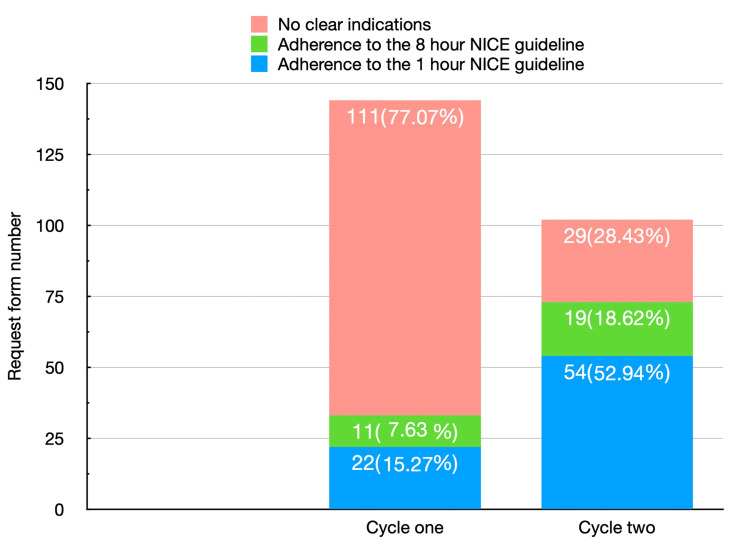
Demonstrates the comparison of CT head scans in adherence to the NICE guidelines, one hour and eight hours, or no clear indications in both cycles. Each column represents a cycle. Each colored portion represents the adherent or non-adherent indication. The data are presented as n (%). NICE: National Institute for Health and Care Excellence.

Regarding adherence to NICE guidelines for one and eight hours separately, Figure 1 shows the number and percentage of head CT scan request forms that meet the NICE criteria for one and eight hours during cycle one and cycle two, along with the non-adherent request forms.

We found 22 (15.27%) of the first cycle scans met the NICE criteria for one hour, while only 11 scans (7.63%) met the NICE criteria for eight hours. In comparison, during the second cycle, 54 scans (52.94%) met the NICE criteria for one hour, and 19 scans (18.62%) met the NICE criteria for eight hours. These findings show significant increases of 37.94% and 10.62% in adherence to the NICE criteria for one hour and eight hours, respectively, after implementing the interventions, with a p-value less than 0.05, as shown in Table 5.

**Table 5:** Demonstrates the specific difference in adherence to NICE criteria for one and eight hours between two cycles. The data are presented as n. Statistical analysis was performed using the Chi-square test, with significance set at p<0.05. Chi-square value (χ²) = 39.67 for documenting one-hour indications; Chi-square value (χ²) = 7.59. for documenting eight-hour indications. NICE: National Institute for Health and Care Excellence.

Cycle	Total scans	Scans with one-hour indications	Scans without one-hour indications	P-value	Scans with eight-hour indications	Scans without eight-hour indications	P-value
Cycle one	144	22	122	<0.0001	11	133	0.0059
Cycle two	102	54	48		19	78	

Regarding the impact of clinical information adequacy in request forms on adherence to NICE guidelines, cycle one revealed that 22 request forms adhering to NICE guidelines contained adequate clinical information, compared to only nine request forms outside the NICE guidelines with sufficient information. This indicates a significant association with a p-value less than 0.05. Consequently, promoting proper completion of radiology request forms was adopted as one of the interventions. Table 6 illustrates this association.

**Table 6:** Demonstrates the association between the adequacy of clinical information in request forms and the adherence to NICE guidelines in cycle one. The data are presented as n. Statistical analysis was performed using the Chi-square test, with significance set at p<0.05. Chi-square value (χ²) = 51.63 NICE: National Institute for Health and Care Excellence.

Adequacy of clinical information in cycle one	Scan numbers	Scans in adherence to NICE guideline	Scans with no clear indication	P-value
Scans with fully adequate clinical information	31	22	9	<0.0001
Scans with inadequate clinical information	113	11	102	

The details of indications for CT head scans according to NICE guidelines for one-hour and eight-hour criteria during cycle one and cycle two are shown in Table 7.

**Table 7:** The documented one-hour and eight-hour indications for head CT after trauma in both cycles. Indications documented are presented as n (%). Each column (cycle one or cycle two) has its own separate count numbers and percentages of indications, corresponding to either cycle one or cycle two. GCS: Glasgow Coma Scale.

NICE guideline indications	Cycle one (indications documented: 33)	Cycle two (indications documented:73)
Indications for CT head scan within one hour	22 (66.66%)	54 (73.97%)
A GCS score of 12 or less on initial assessment in the emergency department	8 (24.24%)	17 (23.28)
A GCS score of less than 15 at two hours after the injury on assessment in the emergency department	0 (0%)	9 (12.32%)
Suspected open or depressed skull fracture	5 (15.15%)	10 (13.69%)
Any sign of basal skull fracture (hemotympanum, 'panda' eyes, cerebrospinal fluid leakage from the ear or nose, Battle's sign)	5 (15.15%)	8 (10.95%)
Post-traumatic seizure	1 (3.03%)	2 (2.73%)
Focal neurological deficit	2 (6.06%)	3 (4.11%)
More than one episode of vomiting	1 (3.03%)	5 (6.84%)
Indications for CT head scan within eight hours for patients with some loss of consciousness or amnesia	11 (33.33%)	19 (26.02%)
Age 65 or over	7 (21.21%)	11 (15.06%)
Any current bleeding or clotting disorders	0 (0%)	3 (4.11%)
Dangerous mechanism of injury (a pedestrian or cyclist struck by a motor vehicle, an occupant ejected from a motor vehicle or a fall from a height of more than 1 m or five stairs)	4 (12.12%)	5 (6.84%)
More than 30 minutes' retrograde amnesia of events immediately before the head injury.	0 (0%)	0 (0%)

Table 8 shows the indications mentioned that were not adherent to the NICE guidelines in both cycles.

**Table 8:** Documented indications that are not adherent to the NICE guidance. Indications documented are presented as n (%). NICE: National Institute for Health and Care Excellence.

Indications	Cycle one (indications documented: 111)	Cycle two (indications documented: 29)
No specific indication mentioned apart from head trauma	53 (47.74%)	13 (44.82%)
Fall from height (not mentioned how height)	17 (15.31%)	4 (13.79%)
Road traffic accident (not specific)	21 (18.91%)	7 (24.13%)
Fall on ground	4 (3.6%)	2 (6.89%)
Vomiting (frequency not mentioned)	6 (5.4%)	3 (10.34%)
Altered mental state (not specific term)	10 (9.01%)	0 (0%)

## Discussion

During cycle one of the audits, it was observed that 77% of patients presenting with head trauma had CT head scans outside the NICE guidelines, indicating significant overutilization of the scan and unnecessary radiation exposure. This rate is higher than in a similar study conducted at a teaching Hospital in Basrah, Iraq, where 59% of pre-intervention CT scans did not adhere to NICE guidelines [18]. This difference may be due to crowding, as doctors in a busier emergency department (144 patients with head trauma at the Teaching Hospital in Baghdad compared to 59 at the Teaching Hospital in Basrah) had less time per patient and adopted a strategy of scanning all patients with CT head scans to avoid missing any clinically significant brain injuries.

Furthermore, we found that insufficient clinical information provided by ED doctors on request forms led to excessive head CT scan requests outside the NICE guidelines during cycle one, with a significant association indicated by a p-value less than 0.05. Additionally, it underestimated adherence because clinicians did not document clinical information accurately. As a result, promoting the completion of radiology request forms has been adopted as one of our interventions in cycle two.

Many non-clinical factors influence a provider's decision to order a CT scan in patients with minor head injuries, which were not measured in this study but are discussed in the literature. These include patient expectations, anxiety, fear of litigation and missed diagnoses, desire to expedite diagnosis, increased availability of CT, and defensive medical practices due to liability threats (especially for ED physicians) [6,19]. Additionally, difficulties in changing practice models, resistance from colleagues, and a lack of trust in evidence may also play a role [20].

It was observed that most indications not adhering to the NICE guidelines were non-specific terms, which were insufficient to justify the performance of the CT scan. In cycle one, 53 request forms mentioned only head trauma with no specific complaints. Clear risk factors should accompany head trauma to justify the benefit of a CT scan over radiation exposure. The second most common indications were mechanisms of injury documented in 42 request forms. The mechanism of injury (like RTA or fall from height) should also include the severity to justify the head CT scan according to NICE guidelines (such as a pedestrian or cyclist struck by a motor vehicle, an occupant ejected from a motor vehicle, or a fall from a height of more than 1 m or five stairs).

After implementing our interventions, we observed significant improvements in the system. There was a 33% reduction in the total number of CT head scans per month, indicating that scans were avoided for patients with minor head injuries who had no indications according to NICE guidelines. Additionally, we substantially increased the proportion of head CT scans performed with indications matching NICE guidelines for both one-hour and eight-hour criteria, rising from 22.91% to 71.56%. These results are similar to those seen in cycle two of the study conducted at the Teaching Hospital in Basrah, Iraq, and demonstrate that our interventions have successfully enhanced the appropriate use of CT head scans for head trauma in accordance with NICE guidelines.

Although we did not achieve our target of complete compliance with the NICE guidelines, we succeeded in reaching 71.56% in compliance with the NICE guidelines and avoiding unnecessary CT scans for minor brain injury cases with no clear risk factors. These interventions improved clinical practice in the emergency department by avoiding unnecessary scans, reducing emergency overcrowding and resource wastage, and providing more attention to patients who might have a significant clinical event. Consequently, maintaining these modifications and implementing additional strategies is essential to attain the desired quality objective, as there is always room for improvement.

Limitations 

The study conducted in a busy emergency department highlights the need to sustain efforts in maintaining the implemented intervention, which involves using new request forms with a checklist. These efforts should focus on ensuring the adequacy of clinical information in the request forms and educating ED doctors and staff. This requires an ongoing commitment from the relevant departments, including follow-up on our interventions and feedback to ED doctors. Maintaining the use of the checklist request form and education sessions is essential to keep and improve the practice of requesting the head CT scan.

In cycle one, we found that request forms with inadequate clinical information often contained poorly documented indications that did not justify the performance of CT head scans. However, this does not mean that some patients did not need the CT scan; it may simply be due to poor documentation that led to missing the indications. Furthermore, although improving the adequacy of clinical information was one of our interventions and contributed to better documentation of indications in cycle two, it is difficult to isolate the effect of just improving the completion of radiology request forms in cycle two, as other interventions were also implemented that could have influenced the results. Nevertheless, the benefits of adequate clinical information are well supported by numerous studies, as they help justify the need for CT scans and assist radiologists in improving the accuracy of their reports [21].

This study was conducted in one of the major hospitals in Baghdad and is considered a single-center study; therefore, its generalizability to other hospitals in Iraq may be limited. The study duration met the duration recommended by the Royal College of Radiologists for such studies; however, its duration is considered short to follow up on the maintenance of the changes.

## Conclusions

This clinical audit improved the use of CT head scans for patients presenting with head trauma in the emergency department by aligning with NICE guidelines for initial head trauma management. It helps prevent unnecessary CT scans when clinical indications do not justify radiation exposure and reduces waste of hospital resources and crowding in the emergency department. It is essential to sustain the improvements by following up on the interventions after the study and continuing to provide education sessions about the NICE guidelines and the new checklist request forms. This thereby enhances practice and achieves 100% adherence. Maintaining the current level of quality improvement will require additional audits to ensure ongoing quality assurance.
